# ASO Author Reflections: Age at Oncological Surgery Affects the Long-Term Risk of Being Bedridden in Elderly Patients

**DOI:** 10.1245/s10434-023-13583-4

**Published:** 2023-05-04

**Authors:** Takaaki Konishi, Yusuke Sasabuchi, Masahiko Tanabe, Yasuyuki Seto, Hideo Yasunaga

**Affiliations:** 1 Department of Breast and Endocrine Surgery, Graduate School of Medicine, The University of Tokyo, Tokyo, Japan; 2 Department of Clinical Epidemiology and Health Economics, School of Public Health, The University of Tokyo, Tokyo, Japan; 3 Data Science Center, Jichi Medical University, Tochigi, Japan

## Past

The prevalence of malignant tumors is increasing, especially in elderly patients aged ≥ 65 years, and they often become candidates for oncologic surgery.^1^ Impaired activity of daily living following surgery is a serious concern in the indication because poor functional capacity can result in several complications and a short survival prognosis.^2^ Furthermore, many elderly patients prioritize functional prognosis over survival prognosis.^3^ However, there has been little evidence regarding the long-term functional prognosis following oncologic surgery.

## Present

We retrospectively investigated the association between age at oncologic surgery and the postoperative incidence of bedridden status and mortality among 11,896 patients aged ≥ 65 years, using a Japanese administrative database.^4^ Multivariable survival analysis with adjustments for patient backgrounds and treatment courses showed that patients aged ≥ 70 years had a significantly higher incidence of being bedridden than those aged 65–69 years within a median follow-up period of 588 (interquartile range, 267–997) days; the sub-distribution hazard ratios of the age groups of 70–74, 75–79, 80–84, and ≥ 85 years were 3.20 (95% confidence interval, 1.53–6.71), 3.86 (1.89–7.89), 6.26 (3.06–12.8), and 8.60 (4.19–17.7), respectively. Patients aged ≥ 75 years had significantly higher mortality than those aged 65–69 years; the hazard ratios of age groups of 75–79, 80–84, and ≥ 85 years were 1.75 (1.44–2.14), 2.63 (2.16–3.22), and 3.77 (3.07–4.65), respectively.

## Future

Our study revealed that age is a crucially important predictor for long-term functional and survival outcomes irrespective of the surgery site and preoperative performance status. The current study can scientifically provide useful information on the preoperative decision-making process. Moreover, because the hazard ratio for being bedridden increased rapidly compared with that for mortality, long-term observation and maintenance of activities (e.g., rehabilitation and care intervention^5^) following oncologic surgery should be generally highlighted for elderly patients.
